# Eco-Environmental, Health and Socio-Economic Perspectives on Cyanobacteria and Cyanotoxins

**DOI:** 10.3390/toxins18100456

**Published:** 2026-10-20

**Authors:** Liang Chen, Jun Chen

**Affiliations:** 1 Qilu Lake Field Observation and Research Station for Plateau Shallow Lake in Yunnan Province, Yunnan Key Laboratory of Ecological Protection and Resource Utilization of River-Lake Networks, Institute for Ecological Research and Pollution Control of Plateau Lakes, School of Ecology and Environmental Science, Yunnan University, Kunming 650500, China; 2 State Key Laboratory of Vegetation Structure, Function and Construction (VegLab), Yunnan University, Kunming 650500, China; 3 Southwest United Graduate School, Kunming 650092, China; 4 Donghu Experimental Station of Lake Ecosystems, Institute of Hydrobiology, Chinese Academy of Sciences, Wuhan 430072, China; 5 University of Chinese Academy of Sciences, Beijing 100049, China

**Keywords:** blue-green algae, cyanobacterial harmful algal blooms, taste and odor, cyanobacterial toxins, cyanobacterial biomass–toxicity paradox, 3P framework

## Abstract

Cyanobacteria, or blue-green algae, the first oxygenic photosynthetic organisms on Earth, play indispensable ecological roles as primary producers and drivers of biogeochemical cycles, and are also sources of human food, nutraceuticals, and biotechnological products. However, cyanobacterial harmful algal blooms (CyanoHABs), intensified by eutrophication and global climate change, threaten water quality, biodiversity, and human health due to their production of diverse secondary metabolites, i.e., taste and odor (T&O) compounds and cyanotoxins. This Special Issue, “Ecology and Toxicology of Cyanobacteria and Cyanotoxins”, compiles 13 papers from 9 countries that collectively advance our understanding of these issues across multiple scales. Cyanotoxins, including microcystins, cylindrospermopsins, anatoxins, and saxitoxins, can cause various hazards to animals and humans, including systemic toxicity, hepatotoxicity, genotoxicity, carcinogenicity, reproductive/developmental toxicity, neurotoxicity, immunotoxicity, and endocrine disruption. CyanoHABs are governed by complex bottom-up and top-down regulation, and blooms and cyanotoxin production are closely linked, but not always consistent/synonymous. Environmental stressors, e.g., phosphorus limitation, cold shock, and grazing pressure, selectively favor toxigenic genotypes, or can increase the cellular cyanotoxin quota, although overall population growth is suppressed. This can be explained by the fact that cyanotoxins can help cyanobacteria to better adapt to environmental stressors. When blooms disperse, demise, or are disrupted by certain interventions, the reduced biomass may increase exposure to dissolved, extra-cellular cyanotoxins if intra-cellular toxins are released faster than they are degraded or removed. We term the uncoupling of biomass and toxicity as the “cyanobacterial biomass–toxicity paradox”. We propose a “3P framework”, i.e., prediction, prevention, and prioritization, for the evidence-based risk management of CyanoHABs and cyanotoxins. We also advocate for more studies using multi-omics and artificial intelligence, as well as multi-disciplinary, multi-sectoral, and multi-stakeholder collaboration under the “One Health” umbrella to translate science into actionable solutions for sustainable development.

## 1. Introduction

**Figure 1:**
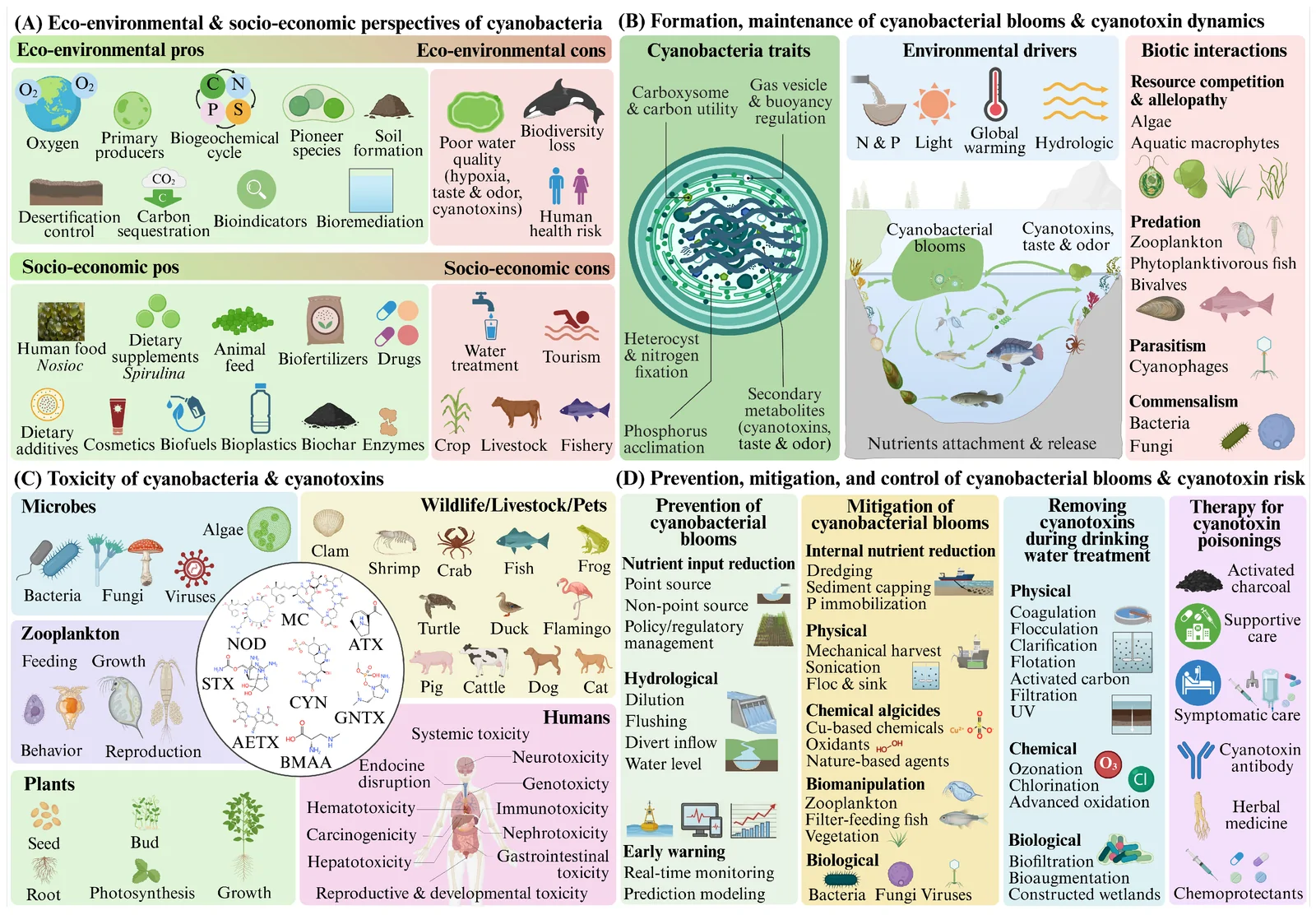
Eco-environmental, health, and socio-economic perspectives on cyanobacteria and cyanotoxins. (**A**) Eco-environmental and socio-economic perspectives on cyanobacteria. Their oxygenic photosynthesis triggered the oxidation of Earth’s atmosphere. Also, cyanobacteria act as primary producers, drive biogeochemical cycles, and serve as pollution bioindicators. Some cyanobacteria have potential applications as animal feed and human food, e.g., *Nostoc flagelliforme* in China, and *Spirulina* spp. (e.g., *Spirulina platensis*) is a worldwide nutraceutical. Cyanobacteria serve as cell factories for producing various value-added products, spanning from high-value drugs, dietary additives, and cosmetics, to high-volume commodities, e.g., biofuels and bioplastics. Cyanobacteria also show potential in pollutant bioremediation. However, harmful cyanobacterial blooms can also cause poor water quality, turbidity, hypoxia, unpleasant tastes and odors (T&Os), cyanotoxins, and threaten biodiversity, water treatment, tourism, fishery, agriculture, and human health. (**B**) Formation and maintenance of cyanobacterial blooms and cyanotoxin dynamics. Several cyanobacterial traits, including gas vesicles and buoyancy regulation, carbon utility, nitrogen fixation, phosphorus acclimation, and secondary metabolite production, e.g., cyanotoxins and T&O compounds, provide them with competitive advantages over eukaryotic phytoplankton. Key environmental factors, including eutrophication and global warming, can intensify cyanobacterial blooms. Cyanobacterial blooms also result from complex biotic interactions, including resource competition and allelopathy with macrophytes, predation by herbivores (e.g., zooplankton, phytoplanktivorous fish, etc.), parasitism by cyanophages, and symbiosis with bacteria. (**C**) Toxicity of cyanobacteria/cyanotoxins to microbes, plants, animals, and humans. Numerous cases of lethal poisoning have been associated with cyanotoxin ingestion in wild animals, livestock, and pets. In humans, few episodes of lethal or severe poisoning have been recorded after acute or short-term exposure, but chronic exposure remains a critical issue, causing various hazards, including hepatotoxicity, genotoxicity, carcinogenicity, and reproductive/developmental toxicity. (**D**) Prevention, mitigation, and control of cyanobacterial blooms and cyanotoxin risk. The prevention methods for cyanobacterial blooms include nutrient input reduction and early warning systems. The mitigation methods for blooms include hydrological methods, internal nutrient reduction, biomanipulation, and physical, chemical, and biological methods. Methods for removing cyanotoxins during drinking water treatment include physical, chemical, and biological methods. Therapy methods for cyanotoxin poisoning including activated charcoal treatment, supportive and symptom-directed care, toxin antibodies, herbal medicine, and chemoprotectants.

Cyanobacteria, or blue-green algae, were the first oxygenic photosynthetic organisms on Earth, evolving ~3.5 billion years ago, and triggered one of the most dramatic events during the evolution of Earth, i.e., the formation of Earth’s oxidizing atmosphere (Figure 1) [1,2,3]. Cyanobacteria, once placed in the kingdom Monera and the division Cyanophyta, are now classified as a phylum within the domain Bacteria and comprise more than 150 genera and 2000 species [4,5]. Today, this primitive group of Gram-negative, bacterial, prokaryotic microorganisms can be found in all terrestrial and aquatic ecosystems, being quantitatively among the most important organisms on Earth, with a conservative estimate of global biomass being 3 × 10^14^ g carbon (C), or a thousand million tonnes (10^15^ g) in wet biomass [1]. As primary producers, cyanobacteria are significant components in the food web and key drivers of biogeochemical cycles of elements, including C, nitrogen (N), phosphorus (P), and sulfur (S). Some cyanobacteria also have potential uses in animal feed and human food [6,7].

**Figure 2:**
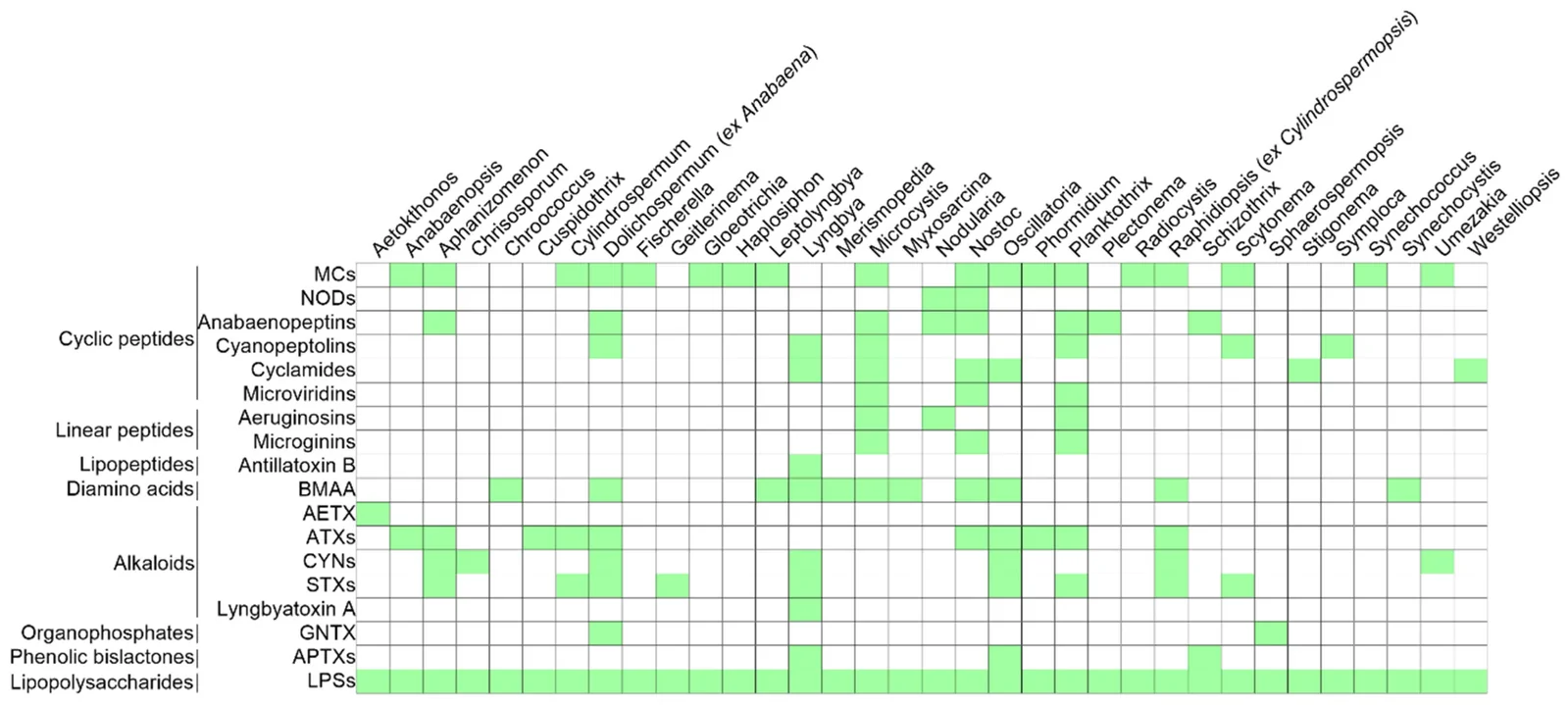
Cyanotoxins and their producing cyanobacterial genera. Data is collected from the references [13,18,19,20,21,22]. AETX, aetokthonotoxin; APTXs, aplysiatoxins; ATXs, anatoxins; BMAA, β-N-methylamino-L-alanine; CYNs, cylindrospermopsins; GNTX, guanitoxin, formerly known as anatoxin-a(S); LPSs, lipopolysaccharides; MCs, microcystins; NODs, nodularins; STXs, saxitoxins. Green cells indicate that the type of cyanotoxin can be produced by the corresponding cyanobacterial genus.

However, eutrophication and global warming are likely to increase the frequency, magnitude, intensity, and duration of cyanobacterial blooms, scums, biofilms, or mats, in many aquatic ecosystems globally (Figure 1) [2,8,9]. Cyanobacterial harmful algal blooms (CyanoHABs) can cause poor water quality, including increased turbidity, hypoxia, anoxia, and the production of unpleasant tastes and odors (T&Os). CyanoHABs are also a potential health hazard, because some species can produce toxic secondary metabolites, i.e., cyanotoxins (Table 1, Figure 1 and Figure 2), e.g., microcystins (MCs) [10,11,12,13,14,15,16,17,18,19,20,21,22], which are toxic to other living organisms, including microbes, plants, animals, and humans [6,23,24,25,26,27].

Significant progress has been made in different aspects regarding the ecology and toxicology of cyanobacteria and cyanotoxins, driven by socio-economic development and advances in science and technology. However, as biology, ecology, and toxicology are very complicated, there are still many ecological and healthy consequences that have not been investigated deeply enough, as well as many gaps. Thus, this Special Issue, “Ecology and Toxicology of Cyanobacteria and Cyanotoxins”, published in the journal *Toxins*, aimed to gather new studies that could contribute to topics related to this issue. This goal was achieved with a compilation of 13 papers (10 research articles and 3 reviews, Table 2) from authors working in 9 countries.

## 2. Eco-Environmental/Socio-Economic Perspectives on Cyanobacteria and the Role of Cyanotoxins

Every coin has two sides. Cyanobacteria represent a classic double-edged sword: cyanobacteria provide crucial ecological services, but their harmful blooms (CyanoHABs) cause poor water quality, turbidity, hypoxia, unpleasant T&Os, and cyanotoxins, and threaten biodiversity, water treatment, tourism, fishery, agriculture, and human health (Figure 1) [1,2,3]. Their oxygenic photosynthetic activity triggered the oxidation of Earth’s atmosphere. Also, cyanobacteria act as primary producers and are a significant component in the food web, driving biogeochemical cycles, while also serving as bioindicators of environmental pollution [28,29,30]. Some cyanobacteria have potential uses as animal feed and human food, e.g., *Nostoc flagelliforme* (hair vegetable, known as “发菜” or “Fa-Cai” in Chinese), *Nostoc sphaeroides* (葛仙米, Ge-Xian-Mi), and *Nostoc commune* (地木耳, Di-Mu-Er) in Asia, especially in China. The cyanobacterial biomass commonly known as “*Spirulina*”, which primarily comprises *Limnospira* (formerly *Arthrospira*) species, including *L. platensis*, *L. maxima*, *L. indica*, and *L. fusiformis*, are dietary supplements worldwide, and *Spirulina subsalsa* is a promising edible resource [6,7]. Cyanobacteria produce various physiologically active and chemically diverse secondary metabolites, including saccharides, lipids, fatty acid amides, polyketides, macrolides/lactones, terpenoids, phenols, cyclic and acyclic peptides, depsipeptides, lipopeptides, alkaloids, vitamins and pigments [7,29,31]. Over 50% of marine cyanobacteria can be used to extract bioactive compounds that can destroy cancer cells through apoptosis [29]. Their effectiveness as anti-tumor, anti-viral, anti-bacterial, anti-fungal, anti-oxidative, anti-inflammatory, anti-aging, and immuno-suppressive drugs and dietary additives is extensively documented [7,29,31]. Recently, cyanobacteria have been encapsulated in sprayable therapeutic hydrogels as living oxygen generators that simultaneously sensitize tumor cells to nitrosative stress-triggered cell death and accelerate wound healing, via secreted extracellular vesicles [32]. Also, cyanobacteria are an environmentally friendly resource for biotechnology, synthetic biology, photobioreactors, cell factories, enzymes, commercial applications, and the production of a wide range of value-added products, spanning from high-value dietary additives, cosmetics, cosmeceuticals, and natural colorants/pigments, to high-volume commodities, biofertilizers, bioplastics, biochar, and future clean energy sources such as biofuel, including biodiesel, bioethanol, and hydrogen [28,29,33,34,35,36]. Cyanobacteria also have potential for the bioremediation of toxic xenobiotics and pollutants [29,33,35,36,37,38], can be engineered into photosynthetic living materials for dual carbon sequestration [39], and can be programmed with conductive pili to enhance photosynthetic electron extraction in biophotovoltaics [40].

Given the high energetic and nutritional costs associated with the biosynthesis of secondary metabolites, cyanotoxins and T&O compounds should confer a selective evolutionary advantage to their producing organisms. In accordance with this, several eco-physiological roles of cyanotoxins and other secondary metabolites have been documented in aquatic ecosystems [41,42,43,44], including: (1) metabolic regulation, including C, N, P and iron (ferrum, Fe) assimilation and metabolism; (2) structural and homeostatic support, including thylakoid integrity, sodium (Na) homeostasis, and oxidative stress adaptation; (3) cellular signaling and communication; (4) developmental and population processes, including programmed cell death, colony formation and maintenance; and (5) ecological interactions, including allelopathy, grazing deterrence, and intra- and inter-specific competition.

Dulić et al. (2022) studied cyanotoxin production in cyanobacteria-dominated biological loess crusts (BLCs) from China, Iran, and Serbia and in BLC-isolated cyanobacterial cultures. Cyanobacteria play important roles in biocrusts, including soil surface stabilization through the immobilization and aggregation of particles, nutrient cycling and storage, and the control of infiltration and runoff. The Biological LOess Crust—DUSt Trapping (BLOCDUST) hypothesis postulates the role of cyanobacterial biocrusts in loess formation and the potential of using cyanobacteria for the restoration of damaged loess surfaces through the development of BLCs. However, no MCs (MC-LR, dmMC-LR, MC-RR, dmMC-RR, MC-LY, MC-LW, MC-YR, or MC-LF), cylindrospermopsins (CYNs), saxitoxins (STXs), gonyautoxin 2-, 3- hydrochloride (GTX2/3), or β-N-methylamino-L-alanine (BMAA) were detected in the biocrusts or cyanobacterial cultures via liquid chromatography-tandem mass spectrometry (LC-MS/MS). Also, biosynthetic genes for MCs (*mcyE*), CYN (*cyrJ*), STX (*sxtA*, *sxtG*, and *sxtS*), and anatoxins (ATXs, *anaC*) were absent in cyanobacterial strains. Thus, cyanotoxin production is not common in BLC and is not a reliable biosignature candidate of cyanobacterial communities in the reconstruction of the paleoclimate and paleoenvironment in loess. However, there might be other (toxic) secondary metabolites present. Further studies are therefore needed to study the eco-physiological roles of cyanotoxins and other secondary metabolites in various terrestrial environments.

## 3. Cyanobacterial Blooms, Cyanotoxins, Environmental Drivers, and Aquatic Communities

Biology and ecology are complicated and nowhere is this complexity more evident than in aquatic systems [45]. On one hand, aquatic environment and communities can affect the occurrence and development of cyanobacterial blooms (Figure 1) [2]. On the other hand, cyanobacterial blooms often cause a cascade of changes in aquatic environments, as well as in planktonic and benthic communities. Several traits of cyanobacteria, including gas vesicles and buoyancy regulation, C utility, N fixation, P acclimation, and secondary metabolite production, e.g., cyanotoxins and T&O compounds, provide them with a distinct competitive advantage over eukaryotic phytoplankton, increasing their dominance and enabling the development of dense cyanobacterial blooms [2,46,47]. Recently, a novel family of surface appendages, cyanobacterial tubular pili, were characterized in *Microcystis aeruginosa* [44]. These pili not only facilitate cell aggregation, colony formation, and buoyancy maintenance, but also enrich MCs and Fe on the cell surface, reinforcing the overall fitness of bloom-forming cyanobacteria. Key environmental factors, including eutrophication, rising CO_2_ concentrations, increased temperature and global warming, extreme wet–dry cycles, hydrologic events, and chemical contaminants are likely to intensify cyanobacterial blooms [48]. Cyanobacterial blooms also result from complex interactions within the aquatic ecosystem, e.g., resource competition and allelopathy with aquatic macrophytes and other algae, grazing by herbivores, including zooplankton, phytoplanktivorous fish, and bivalves, parasitism by cyanophages, and symbiosis with bacteria and fungi [2,30,49]. In other words, the formation and maintenance of cyanobacterial blooms can be conceptualized within the classic ecological framework of bottom-up and top-down regulation [8,47,49]. Bottom-up regulation refers to control by resource availability, primarily nutrients, CO_2_, and light, which determine the potential carrying capacity of cyanobacterial populations. This is the mechanistic basis for nutrient reduction strategies in bloom management (Section 5). Top-down regulation, in contrast, refers to control by consumers, including zooplankton, filter-feeding fish and bivalves, which can suppress cyanobacterial biomass regardless of resource availability [8,50,51].

Hu et al. (2021) investigated the compositions of bacterial and viral communities in the Macau Storage Reservoir (MSR), Macao Special Administrative Region (SAR), China, between 2013 and 2016, by means of 16S rRNA gene sequencing and metagenomics. In recent years, MSR, the main storage reservoir for drinking water in Macau, has been experiencing eutrophication with blooms of algae and cyanobacteria, dominated by *M. aeruginosa* and *Raphidiopsis raciborskii* (formerly *Cylindrospermopsis raciborskii*), which can produce MCs and CYNs, respectively. The maximum algal density was about 2 × 10^8^ cells/L. The most prevalent phyla of bacterial communities were proteobacteria (32%), Cyanobacteria (18.9%), Bacteroidetes (14.1%), and Verrucomicrobia (11.5%). Cyanobacteria were composed primarily of species from five genera, i.e., *Raphidiopsis*, *Synechococcus*, *Oscillochloris*, *Microcystis*, and *Cyanobacterium*. The dominant family of viruses was Phycodnaviridae, which accounted for 70% of the total. The primary viruses included Organic lake *Phycodnavirus* 2, an unidentified *Phycodnavirus* 1, Yellowstone lake *Phycodnavirus* 1, *Phaeocystis pouchetii* virus 1, *P. pouchetii* virus, *Phaeocystis globosa* virus, and uncultured Phycodnavirus. Algal density was statistically positively related to Phycodnavirus species number. However, there was no significant relationship between the relative abundance of cyanobacteria and Phycodavirus species number. A positive relationship was observed between the relative abundances of *Raphidiopsis* and Proteobacteria. Also, redundancy analysis (RDA) showed that the concentrations of total nitrogen (TN), ammonia-nitrogen (NH_4_-N), and total phosphorus (TP), pH and temperature were associated with cyanobacterial and viral communities. However, the exact mechanisms of interaction between cyanobacteria, viruses, and environmental factors remain unclear.

It should be noted that, cyanobacterial blooms and cyanotoxin production are closely linked, but not always consistent [24,25,26,47]. In other words, cyanotoxin risk cannot be inferred from cyanobacterial biomass alone. While blooms represent excessive biomass accumulation, toxin production depends on both the presence of toxigenic genotypes and the physiological state of cyanobacterial cells. Importantly, toxigenicity is often intertwined with eco-physiological traits, such as nutrient acquisition and trophic state adaptation [47], and the ability to produce cyanotoxins may be part of a specific ecological niche rather than a standalone trait. Generally, cellular toxin quotas typically scale with growth rate, reflecting a coupling between primary metabolism and secondary metabolism. However, this coupling is not universal. For example, P limitation can lower cyanobacterial biomass but make N and light more available, which can favor toxigenic cells over nontoxigenic ones, and increase MC concentrations [24]. Both field data from Lake Erie and multi-strain laboratory experiments demonstrate that N availability shapes MC variant profiles via a stoichiometric trade-off: higher N promotes N-rich, but low-toxicity MC-RR, whereas N limitation induces N-poor, but more toxic MC-LA and MC-LW [52,53]. Hence, reducing N inputs may lower total MCs but paradoxically increase toxic risk by increasing the potent variants. Furthermore, toxin production was increased after exposure to herbivorous zooplankton [54], phytoplanktivorous and omnivorous fish [55,56], or cooler temperatures [57,58], irrespective of biomass accumulation. Thus, environmental stressors, e.g., P limitation, cold shock, and grazing pressure, selectively favor toxigenic genotypes, or increase toxin production per cell, although overall population growth is suppressed. This can be explained by the fact that MCs can help cyanobacteria to better adapt to environmental stressors. We term the uncoupling of cyanobacterial biomass and cyanotoxin toxicity the “cyanobacterial biomass–toxicity paradox”. Also, bloom dynamics and toxin dynamics are not synonymous, and the success of cyanotoxin treatment cannot be judged solely by bloom suppression [26]. When blooms mix, disperse, demise, lyse, or are disrupted by certain interventions, the reduced biomass may increase exposure to dissolved, extra-cellular cyanotoxins if intra-cellular toxins are released faster than they are degraded or removed. Such uncoupling of cyanobacterial biomass and cyanotoxin toxicity has profound implications for risk assessment, as bloom intensity alone is not a reliable predictor of toxic risk. Instead, the cyanotoxin risk can be calculated based on cyanotoxin type, toxic equivalency factor (TEF), and toxigenic cell number according to the following equations:

(1) $$\mathrm{Total}\;\mathrm{cyanotoxin}\;\mathrm{toxicity}=\sum_{i=1}^{n}\mathrm{Cyanotoxin}\left(i\right)\;\mathrm{concentration}\times \mathrm{TEF}\;(\mathrm{Cyanotoxin}\;i)$$

Total cyanotoxin concentration = Intra-cellular cyanotoxin concentration + Extra-cellular cyanotoxin concentration(2)

Intra-cellular cyanotoxin concentration = Cyanotoxin quota × Toxigenic cyanobacteria cell density(3)

Another point is that research on benthic cyanobacteria and their toxins is urgently needed, because cyanobacterial mats are increasingly reported worldwide, produce virtually all classes of cyanotoxins, and exhibit extreme spatiotemporal variability that renders conventional monitoring inadequate [47,59,60]. Also, the drivers of different cyanobacteria and cyanotoxins are not the same [47,49]. For example, *Microcystis* and MC biosynthesis *mcy* genes occur globally, with no evidence of geographic isolation, while *Raphidiopsis* is geographically more restricted, and CYN biosynthesis *cyr* genes are only detected in Europe, Australia, and Asia [47]. *Microcystis* can reach high abundance in eutrophic waters, with cell abundance generally paralleled by *mcy* genes, while *Raphidiopsis* abundance shows little correlation with nutrient concentrations but exhibits positive relationships with dissolved oxygen, water temperature, and pH. For toxic Microcystaceae (*Microcystis*, *Aphanothece*, *Aphanocapsa*), *Dolichospermum*, *Chroococcus*, and *Coelosphaerium*, a negative dependence on density for net growth rate was observed, while environmental factors exhibit taxon-specific non-linear effects [49]. Also, the effects of biotic factors on cyanobacterial realized niches vary in a taxon-specific manner across temperature and phosphate gradients. Thus, in studies of cyanobacterial blooms and cyanotoxin dynamics, we encourage more focus on the phenotypes driven by the environment, biogeochemical processes (nutrient pools, metabolites, etc.), and interactions within aquatic communities, including microbial consortia (microbiome) and cyanosphere, as well as the role of chemical communications or chemo-ecological interactions, i.e., cyanobacterial chemical ecology [61]. We believe that state-of-the-art sequencing and omic approaches, including meta-genomics, environmental DNA (eDNA), meta-transcriptomics, meta-proteomics, and metabolomics, as well as artificial intelligence (AI) and machine and deep learning tools, will help to decipher the drivers of undesirable CyanoHABs.

## 4. Toxicity of Cyanobacteria/Cyanotoxins and Human Health

Toxins produced by cyanobacteria, referred to as cyanotoxins, or cyanobacterial toxins, can be categorized according to two main characteristics: (1) their modes of toxicity to multi-cellular animals, animal cells, or cell systems; and (2) their chemical composition and structure [13,62]. Based on their modes of toxicity, cyanotoxins can be classified as: (1) hepatotoxins; (2) neurotoxins; (3) cytotoxins; (4) dermatoxins; and (5) irritants (Table 1). Based on the structure of their molecules, cyanotoxins can be classified into several groups (Table 1, Figure 2): (1) cyclic peptides, including MCs, nodularins (NODs), anabaenopeptins, cyanopeptolins, cyclamides, and microviridins; (2) linear peptides, including aeruginosins and microginins; (3) lipopeptides, including antillatoxins, jamaicamides and kalkitoxins; (4) non-proteinogenic diamino acids, including BMAA, β-N-amino-L-methyl alanine (BAMA), N-(2-aminoethyl)glycine (AEG), and 2,4-diaminobutyric acid (DAB); (5) alkaloids, including CYNs, ATXs, STXs, also known as paralytic shellfish toxins (PSTs), and aetokthonotoxin (AETX); (6) an organophosphorus compound, guanitoxin (GNTX, formerly known as anatoxin-a(S)); (7) phenolic bislactones, aplysiatoxins (APTXs); and (8) lipopolysaccharides (LPS) [12,14,20,63,64,65]. Cyanotoxins can cause toxicity to microbes, plants, animals, and humans (Figure 1) [6,13,66,67]. Numerous cases of lethal poisoning have been associated with cyanotoxin ingestion in wild animals, livestock, and pets [13]. In humans, few episodes of lethal or severe poisoning have been recorded after acute or short-term exposure, but chronic exposure via contaminated drinking water, air, or food remains a critical issue, causing various hazards, including systemic toxicity, hepatotoxicity, genotoxicity, carcinogenicity, reproductive/developmental toxicity, neurotoxicity, immunotoxicity, and endocrine disruption.

Jiang et al. (2022) exposed tadpoles of American bullfrogs (*Lithobates catesbeianus*) at the G26 stage (10 days post fertilization) to 0.5 or 2 μg MC-LR/L for 30 days. MCs caused a dose-dependent increase in the number of internal gill filament epithelial cells or eosinophils. Immunoglobulins (IgA, IgG, and IgM) contents in gills were significantly increased, while lysozyme activities were decreased, and the abilities of gill supernatants to inhibit pathogenic bacteria (*Escherichia coli*, *Pseudomonas aeruginosa*, and *Staphylococcus aureus*) were decreased. Superoxide dismutase (SOD) and caspase 3 activities as well as glutathione (GSH) contents were decreased, while reactive oxygen species (ROS), LPS endotoxin, and interleukin-1β (IL-1β) contents were increased. Transcriptomics identified 706 differentially expressed genes (DEGs) in the 0.5 μg MC-LR/L group, including 249 up-regulated genes (URGs) and 457 down-regulated genes (DRGs), while 666 DEGs were identified in the 2 μg MC-LR/L group, including 213 URGs and453 DRGs. These DEGs were related to gill tight junctions, inflammation, immunity, hypoxic stress, oxygen transport, oxidative stress, and apoptosis. After MC-LR exposure, α-diversity indices (Sobs, Chao, Shannon) of gill microbiota were decreased, while the Simpson index was increased. Analysis of similarity (ANOSIM) and principal coordinate analysis (PCoA) showed that MC-LR affected bacterial community composition.

Zhan and Gong (2024) exposed nematodes (*Caenorhabditis elegans*) to 100 μg MC-LR/L. MC-LR decreased nematodes’ lifespan, egg-laying capacity (brood size), and the hatching rate of eggs. MC-LR decreased the chemotaxis of worms towards benzaldehyde and calcium response in the AWC neurons, suggesting that MCs impaired odor perception functions. Also, this chemotaxis effect was transmitted to the F3 generation (chemotaxis index: F0 < F1 < F2 < F3 < F4, without significant changes in F4), although eggs of F0 and subsequent offspring were not exposed to MCs. The LC-MS/MS results showed MC-LR accumulation in the nematodes, and immunoblotting analysis showed predominant MC-LR accumulation in the head region, which is rich in neurons, but not in the intestine. Further immunoprecipitated assays identified MC-LR-LET-92 complexes, suggesting that MC-LR could interact with LET-92, a homologous protein of protein phosphatase (PP2A) C subunit in the *C. elegans*. Following MC exposure, PP2A activities or chemotaxis in the *let-92(L252A)* and *let-92(C278G)* mutants were not significantly affected, while the *let-92(I132V)* variant exhibited reduced PP2A activities. Also, no MC-LR-LET-92 complexes were detected in the *let-92(L252A)* and *let-92(C278G)* mutants. L252 and C278 formed a binding-site pocket via van der Waals forces and covalent bonds to interact with MC-LR, respectively.

Saha et al. (2022) reported that CYN caused gut microbiome alteration and its connection to hepatotoxicity via the gut–liver axis. They exposed male C57BL/6J mice to 60 µg CYN/kg body mass (bm)/day for 15 days. A decreased Shannon diversity of the gut microbiome (“second brain”) and relative abundance of the phyla Bacteroidetes and Verrucomicrobia, but increased relative abundance of the phyla Firmicutes and Actinobacteria, as well as Firmicutes/Bacteroidetes (F/B) ratio, were observed. At the species level, an increased abundance of the opportunistic pathogen *Clostridioides difficile* and decreased abundance of probiotic flora including *Akkermansia muciniphila*, *Bacteroides thetaiotaomicron*, *Blautia coccoides*, and *Roseburia* were observed. The tight junction (TJ) protein Claudin 2 was up-regulated, while Occludin was down-regulated in the small intestine, which might cause a “leaky gut” phenotype, with a concomitant increase in pathogen-associated molecular pattern (PAMP) endotoxins in the serum (endotoxemia) and damage-associated molecular pattern (DAMP) high-mobility group box 1 (HMGB1) in both the small intestine and serum. Therefore, CYN-induced alteration in the gut microbiome (“gut dysbiosis”) might cause gut leaching, with an increase in circulatory PAMP and DAMP levels, which led to hepatic inflammasome activation, fibrosis, and injury.

Indeed, hematoxylin and eosin (H&E) staining of livers showed increased rarefication of the cytoplasm and vacuolation, and picrosirius red staining showed mild fibrosis and collagen deposition (Saha et al., 2022). Both mRNA and protein expressions of clusters of differentiation 68 (CD68), a Kupffer cell (KC) activation marker, and α-smooth muscle actin (α-SMA), a hepatic stellate cell (HSC) activation marker, were up-regulated. In the liver, HMGB1 and its canonical receptors, receptors for advanced glycation end products (RAGE) and Toll-like receptor 4 (TLR4), as well as its common adapter molecule myeloid differentiation primary response protein (MyD88), were all up-regulated. Gut-derived endotoxins, transported to the liver by the portal vein, can act as the initial signal for the hepatic inflammasome activation by increasing NLR family pyrin domain containing 3 (NLRP3) expression. A second signal, mediated by DAMPs, can further trigger the hepatic inflammation activation. In other words, the NLRP3 inflammasome can be activated by a two-signal model: (1) PAMPs, through the pattern recognition receptor (PRR), TLR4, which can identify endotoxins; and (2) DAMPs. Interestingly, co-localization of the NLRP3 inflammasome with its adapter ASC2 was increased. Also, the pro-inflammatory cytokines IL-1β and IL-6 were up-regulated, and the inflammation might cause hepatocyte apoptosis. Intrinsic apoptosis pathway markers, including pro-apoptotic Bax/Bcl-2 ratio, initiator cleaved Caspase 9, executioner cleaved Caspase 3, and late-apoptotic cleaved Caspase-mediated poly adenosine diphosphate-ribose polymerase (PARP), were all up-regulated. Further results also showed that transforming growth factor beta (TGF-β) might mediate activation of HSCs and Smad2/3-Smad4-mediated fibrosis in liver.

Swartzendruber et al. (2022) reported that cyanobacterium *Geitlerinema* sp. LPS caused neutrophil-dependent lung inflammation and glucocorticoid-resistant asthma, although *Geitlerinema* sp. LPS is not as potent as *E. coli* LPS. Leukocyte infiltration was observed in lungs of male/female C57Bl/6 mice at 24 h after intranasal exposure to 1 µg *Geitlerinema* sp. LPS/g, bm. Diff-Quick staining of bronchial alveolar lavage (BAL) fluid (BALF) showed that the absolute number and percentage of inflammatory neutrophils were increased, while the absolute number and percentage of macrophages were decreased, and there were no significant changes in the number/percentage of eosinophils or lymphocytes. Flow cytometry assay also showed an increased neutrophil (CD11b^+^Ly-6G^+^ cells) number, but decreased F4/80^+^CD11b^+^ macrophage number in the BAL. Contents of chemokines including keratinocyte-derived chemokine, the murine equivalent of IL-8, and RANTES, as well as pro-inflammatory cytokines including IL-6, tumor necrosis factor-alpha (TNF-α), and interferon-gamma (IFN-γ), were increased, while the contents of chemokines macrophage inflammatory protein-2 (MIP-2), the pro-inflammatory cytokines IL-17a, and the anti-inflammatory cytokines IL-10 showed no significant changes. *Geitlerinema* LPS also increased intercellular adhesion molecule-1 (ICAM-1) contents, suggesting that LPS not only increased chemokine production to recruit neutrophils but also up-regulated adhesion molecules, which promoted neutrophil extravasation from the blood to the lung airways. In vitro results also showed that *Geitlerinema* LPS increased ICAM-1 contents in human lung adenocarcinoma airway epithelial A549 cells. IL-6 and IL-8 production in A549 cells was also increased, but the nuclear factor kappa-B (NF-κB) inhibitor, Bay 11-7085, blocked the promotion, suggesting that *Geitlerinema* LPS acted via the NF-κB pathway.

Yang et al. (2023) reported the hepatopancreatic transcriptome and gut microbiome in the gastropod *Bellamya aeruginosa* exposed to MC-producing *M. aeruginosa* FACHB 905 for 1, 3, 7, or 14 days. Compared with green algae *Chlorella vulgaris* NMBlud2006-2, *M. aeruginosa* increased mortality, especially at days 2–4 and 9–10. MC contents in the hepatopancreas of *B. aeruginosa* exposed to *Microcystis* first increased and peaked at day 3, but then decreased. Overall, 242, 429, 402, and 136 URGs were identified on the 1st, 3rd, 7th, and 14th days, respectively, while 258, 290, 320, and 137 DRGs were identified. These DEGs were mainly related to stress responses, xenobiotic biodegradation, immunity, and energy metabolism. The Shannon and Simpson indices of the gut microbiota increased at day 14, whereas the Chao1 and Ace indices showed no significant changes. ANOSIM, PCoA, and hierarchical clustering analysis (HCA) showed an altered intestinal microbial community. The phyla Campylobacterota, Firmicutes, and Myxococcota displayed increased abundance, while Cyanobacteria, Fusobacteriota, and Halobacterota displayed decreased abundance. The co-occurrence network showed a more complex structure caused by *Microcystis*, with increased network nodes, edges, average connectivity (avgK), average clustering coefficient (avgCC), and occupancy of the negative correlations.

In another study (Liu et al., 2023) by the same research group led by Prof. Dr. Jin-Yong Zhu, a similar experimental design and results to Yang et al. (2023) were reported. Liu et al. (2023) exposed *B. aeruginosa* to non-MC-producing *M. aeruginosa* FACHB-469 or MC-producing *M. aeruginosa* FACHB 905 for 7 or 14 days. MC contents in hepatopancreas of *B. aeruginosa* exposed to *M. aeruginosa* FACHB 905 at day 7 were greater than those at day 14. The Chao1, Simpson, and Shannon indices of the gut microbiota were greater in MC-producing groups than in non-MC groups, and were increased from day 7 to 14. Permutational multivariate analysis of variance (PERMANOVA), ANOSIM, and the multi-response permutation procedure (MRPP) showed no significant changes in intestinal microbial composition at day 7, but changes were observed at day 14. The relative abundance of the cellulase-producing bacteria *Acinetobacter* was decreased at day 14, while that of the MC-degrading bacteria *Pseudomonas* and *Ralstonia* was increased. The co-occurrence network showed a more complex structure caused by FACHB 905 and MCs, and effects were more evident at day 14. Network nodes clustered around *Acinetobacter* increased from day 7 to 14 in non-MC groups, and MCs caused the interactions between *Pseudomonas* and *Ralstonia* and other bacteria to shift from positive correlations at day 7 to negative correlations at day 14. The mean nearest taxon distance (MNTD) analysis showed that the community assembly was dominated by stochastic processes in non-MC groups, although the percentage of stochastic processes decreased from 90% at day 7 to 70% at day 14, and MCs triggered the community assembly from stochastic processes (60%) at day 7 to deterministic processes (80%) at day 14.

By using the “*Allium* test”, a plant test system, Pesnya et al. (2022) reported that genotoxic effects of cyanobacteria can be detected even at low MC concentrations (0.08–0.228 µg/L) in natural water, which is below the World Health Organization’s (WHO) provisional chronic (lifetime) guideline value for drinking water (1 µg/L). Frequencies of chromosomal aberrations and micronuclei were increased but not statistically significant, suggesting no clastogenic activity. The most common cytogenetic abnormalities were mitotic disturbances, lagging/vagrant chromosomes, c-mitosis, and polyploidy, which were associated with aneugenic activity. Changes in mitotic indices indicate mitotic arrest in the metaphase of the cell cycle. Therefore, genotoxic mechanisms may be associated with chromosome mis-segregation and damage to mitotic spindles. Genotoxic effects, aneugenic activity, and chromosomal instability were associated with cyanobacteria abundance and MC concentrations.

Lin et al. (2021) summarized the in vivo and in vitro studies on the immunotoxicity of pure MCs, cyanobacterial extracts, and MC-producing cyanobacteria in fishes. The immunomodulatory effects depend on the exposure routes, doses, and duration. The immunotoxic mechanisms of MCs include the inhibition of PP1 and PP2A, oxidative stress, immune cell damage, inflammation, and apoptosis. Future studies should focus on adaptive immunity, the hormesis phenomenon, MC variants other than MC-LR, and the combined effects of aquatic microbial pathogens.

Nugumanova et al. (2023) summarized the cyanotoxins with neurotoxic potential and an association with neurodegenerative diseases (NDs), including MCs, NODs, ATXs, CYNs, STXs, GNTX, and BMAA. The major mechanisms of cyanotoxins’ neurotoxicity include: (1) the blocking of key proteins and channels; (2) the inhibition of essential enzymes, including PPs and phosphoprotein phosphatases; and (3) molecular targets including TLR4 and TLR8, participants in neuronal conduction and neuroinflammation. Specifically, BMAA, the non-canonical amino acids (NCAAs), can disrupt protein synthesis through multiple mechanisms: (1) a substrate for human alanyl-tRNA synthetase (AlaRS), BMAA-tRNA-Ala formation, and escaping from the intrinsic AlaRS proofreading activity; (2) inhibition of cognate amino acid activation and AlaRS editing functions; and (3) misincorporation at serine codons during translation. The mistranslation and misfolding of proteins may cause protein aggregates and NDs, including Alzheimer’s disease (AD), Parkinson’s disease (PD), and amyotrophic lateral sclerosis (ALS). Nugumanova et al. (2023) proposed the “Cyanopeptides Hypothesis”, which posits that the mistranslation mechanism may be evolutionarily ancient, initially serving to outcompete neighboring microorganisms during algal blooms. Also, outcompeting gut symbiotic microorganisms may lead to dysbiosis, increased gut permeability, a shift in blood–brain-barrier (BBB) functionality, and eventually, mitochondrial dysfunction in high-energy-demanding neurons.

In 1996, the worst known human poisoning by *Microcystis* and MCs to date occurred in Brazil [68,69,70]. Significant hepatotoxicity, acute liver failure (ALF), and multiple neurological symptoms, including headache, dizziness, vertigo, nausea, vomiting, lethargy, blindness, tinnitus, and deafness, were observed in patients at the Caruaru hemodialysis center, suggesting that *Microcystis* and MCs caused hepatic encephalopathy (HE). In 2015, after exposure to an intense *Microcystis* sp. bloom and MCs, a 20-month-old girl suffered grade II-III encephalopathy, ALF, cerebral edema, and increased ammonia (NH_3_) concentrations in her blood serum [71]. In a recent study, *Microcystis* extracts and MCs were shown to cause HE in rats, i.e., MCs caused toxicity to the liver, the primary target organ of MCs, which caused secondary effects on the brain, via the liver–brain axis [27].

Previous studies showed that MCs can induce metabolic dysfunction-associated steatotic liver disease (MASLD), formerly named non-alcoholic fatty liver disease (NAFLD) or metabolic dysfunction-associated fatty liver disease (MAFLD), including metabolic dysfunction-associated steatohepatitis (MASH), formerly named non-alcoholic steatohepatitis (NASH) [72,73,74]. The lipotoxicity of adipose tissue and alterations in gut microbial functions play a key role in MASLD onset/development, and the multiple parallel hits hypothesis was proposed [75]. MASLD is a multi-systemic clinical condition that presents with a wide spectrum of extrahepatic manifestations, such as obesity, type 2 diabetes mellitus (T2DM), metabolic syndrome, cardio-vascular diseases (CVDs), chronic kidney disease (CKD), extrahepatic malignancies, cognitive disorders, and polyendocrine metabolic ovarian syndrome (PMOS), formerly known as polycystic ovary syndrome (PCOS) [76,77]. MCs can also exacerbate the progression of hepatic steatosis to MASH, fibrotic MASH, advanced fibrosis, liver cirrhosis, and hepatocellular carcinoma (HCC), acting as a second hit [78].

In addition to cyanobacterial blooms and MC production, climate change can also cause other parallel conditions including heat waves, increases in opportunistic pathogens such as *Vibrios*, and antibiotic resistance, which can affect gut microbiota composition. Chatterjee and More (2023) reviewed the multiple climate-change-induced challenges and their human health effects, as well as the concurrent increase in various lifestyle-associated diseases such as CVDs, MASLD, and T2DM, and interactions along the gut–liver–brain axis. In addition to lifestyle, climate change, pathogens, pre-existing diseases, and cyanotoxin mixtures, many other environmental factors, including altitude, ultraviolet light, and hypoxia, all increased the toxicological complexity of cyanobacteria and cyanotoxins [79]. As environmental reservoirs and biological pumps, cyanobacteria can adsorb, absorb, and accumulate other natural and artificial contaminants, including metals, micro- and nanoplastics (MNPs), pesticides, persistent organic pollutants (POPs), per- and polyfluoroalkyl substances (PFAS), and anti-microbial resistance genes (ARGs) [6,80]. This accumulation, mediated largely through the cyanosphere, represents a critical entry point for these contaminants into aquatic food webs, thereby causing additional or synergistic effects to higher trophic levels.

In addition to these various (mixture) toxic effects, there are cases where cyanobacteria produce new toxic substances due to interactions with other natural and artificial pollutants. A pentabrominated biindole alkaloid called AETX is produced by the cyanobacterium (*Aetokthonos hydrillicola*) in the presence of bromide in water [20]. AETX can be transferred to top predators through the food web and has been identified as the causative toxicant for vacuolar myelinopathy in bald eagles.

Also, cyanobacteria are not the only source of cyanotoxins. For example, STXs were initially characterized as products of marine dinoflagellate, including *Alexandrium* sp. [81]. Also, several diatom species can produce BMAA. LPS endotoxins, are structural components of the outer membrane of most Gram-negative prokaryotes, including the Enterobacteriaceae.

Thus, more studies are needed on the toxicology of cyanobacteria and cyanotoxins, including unknown toxins, and effect-directed analysis (EDA) is a useful tool for the identification of key toxicants in environmental complex mixture samples [6]. However, these studies are limited by the scarcity of trained personnel, research facilities, experimental animals, time, and financial resources for conducting ecotoxicology and environmental toxicology experiments on a broad spectrum of cyanotoxins and other traditional and emerging contaminants [82,83]. We believe that new approach methodologies (NAMs), including high-throughput in vitro assays, omics, AI, and machine and deep learning tools, will be useful for better organizing and interpreting data from both past and present, as well as filling data gaps through read-across and unsupervised learning.

## 5. Prevention, Mitigation and Control of Cyanobacterial Blooms and Cyanotoxin Risk

As described in Section 3, the formation and maintenance of cyanobacterial blooms result not only from external influences, e.g., nutrients, light, and climate and hydrological conditions, but also from complex interactions within an aquatic ecosystem, including resource competition and allelopathy with macrophytes and other algae, predation by herbivores, including zooplankton, phytoplanktivorous fishes, and bivalves, parasitism by cyanophages, and symbiosis with bacteria and fungi [2,8,30,50,84,85]. Several strategies have been developed to combat cyanobacterial blooms, which can be categorized into prevention and mitigation (Figure 1) [2,8,46]. The prevention methods for cyanobacterial blooms include: (1) nutrient input reduction, including point and diffuse sources control, as well as policy/regulatory management; (2) hydrologic regulation, including dilution, flushing, divert inflow, and water level manipulation; and (3) early warning systems, including systematic monitoring and prediction modeling. The mitigation methods include: (1) internal nutrient reduction, including dredging, sediment capping, and P immobilization; (2) physical methods, including mechanical harvest, sonication, floc and sink; (3) chemical methods, including copper-based chemicals, herbicides, antibiotics, oxidants, and nature-derived agents; (4) biomanipulation, including filter-feeding organisms, including zooplankton, bivalves and fishes, as well as vegetation restoration; and (5) other biological methods, using cyanobacteria-lysing and growth inhibition microbes, including bacteria, fungi, and viruses. However, it is often difficult and expensive to control outbreaks of blooms, especially in large lakes [46,86,87]. No single intervention strategy is effective even with recent scientific and technological advances. Methods for removing cyanotoxins during drinking water treatment include: (1) physical methods, including coagulation, flocculation, clarification, flotation, activated carbon, filtration, and ultraviolet (UV) radiation; (2) chemical methods, including ozonation, advanced oxidation, and chlorination; and (3) biological methods, including biofiltration and bioaugmentation [4,63,88]. Therapeutic methods for cyanotoxin-associated poisoning and illnesses include activated charcoal treatment, supportive and symptom-directed care, toxin antibodies, herbal medicine, and chemoprotectants [6,89,90].

As described above, algicidal bacteria have promising applications in controlling cyanobacterial blooms. Zhang et al. (2022) analyzed the transcriptome of *Brevibacillus laterosporus* Bl-zj to study the algicidal mechanism against *M. aeruginosa* FACHB 905. *B. laterosporus* was cultured solely or co-cultured with *M. aeruginosa* for 4 days, when the co-cultured medium turned yellow. Scanning electron microscope observation showed that *B. laterosporus* adhered to the surface of *M. aeruginosa*, and *M. aeruginosa* presented antenna-like mucus, began to rupture or displayed deep depressions, and finally, at day 4, the *M. aeruginosa* cell number decreased sharply, and the cell surface shrank and deformed severely, with abundant mucus on and around it. At day 2, 1078 URGs and 917 DRGs of *B. laterosporus* were identified, while there were 1304 URGs and 1104 DRGs at day 4. Quantitative real-time PCR (qRT-PCR) assays of nine genes indicated that the transcriptomic results were reliable. *B. laterosporus* obtained adenosine tri-phosphate (ATP) and energy via degradation of valine, leucine, isoleucine, and fatty acids, and destroyed *Microcystis* cells via efflux pump transporters, secretion of hydrolytic enzymes, antibiotics, proteases, and other secondary metabolites, e.g., prodigiosin, resulting in cyanobacterial death. However, the underlying algicidal mechanisms are not fully known, especially the dynamics. Applying bacteria in bloom control is still associated with many challenges, and there is still a long way to go.

Meanwhile, a microcosm study by Pesnya et al. (2022) showed that different biotic components (zooplankton, amphipods and fish) changed the cyanobacterial communities, MC concentrations, and toxicity. Zooplankton, dominated by the filter-feeder, cladoceran *Daphnia longispina*, and benthic crustacean amphipods *Hyalella azteca* decreased the density/biomass of cyanobacteria, toxin concentrations, and the toxicity, which may be explained by the consumption of cyanobacteria. However, the omnivorous *Oryzias latipes* maintained the cyanobacterial abundance and toxin concentrations, which may be explained by its labile P excretion. More research is needed to study the interactions of biotic components (including zooplankton, zoobenthos, and fish) and cyanobacteria/cyanotoxins.

Biomanipulation represents the application of bottom-up and top-down regulation for management purposes [8]. By increasing populations of grazers (e.g., *Daphnia*) or filter-feeding fish (e.g., silver and bighead carp), managers aim to intensify top-down control to suppress cyanobacterial blooms, which are termed traditional biomanipulation (TB) and non-traditional biomanipulation (NTB), respectively. Recently, a review of 42 experiments revealed substantial reductions in cyanobacterial abundance by silver and bighead carp in 37 (88% of the 42) cases [8]. In particular, in a whole-lake experiment in Lake Donghu, Wuhan, China, increased stock of silver and bighead carp decreased *Microcystis* blooms from a coverage of 87% in 2021 to 0% in 2022. These results highlighted the effectiveness of NTB ecosystem technology (ecotechnology) based on filter-feeding carp in preventing or diminishing cyanobacterial blooms. However, silver and bighead carp feed more effectively on colony-forming *Microcystis* than on filamentous cyanobacteria, leading to an increase in the abundance of small-sized algae [8]. More studies are needed on methods for controlling blooms of cyanobacteria and other algae/phytoplankton and improving water quality.

More recently, an intrinsic mechanism underlying the abrupt collapse of cyanobacterial blooms was uncovered by a field investigation in Lake Dianchi, Kunming, China and lab experiments on *M. aeruginosa* FACHB 905 [9,91]. During a *Microcystis* bloom demise event, cellular labile Fe overload triggers lipid peroxidation, generating truncated phospholipids that destabilize plasma membranes and induce nanoscale pore formation, ultimately causing ferroptosis in individual cells. Crucially, oxidized lipids released from ferroptotic cyanobacterial cells propagate lipid peroxidation to neighboring cells, thereby spreading death throughout the population. These findings suggest a potential strategy for bloom control: it is possible to actively drive collective cell death by regulating active lipid peroxides.

Here, we propose that effective management of cyanobacterial blooms and cyanotoxin risks hinges on three interconnected pillars, i.e., prediction, prevention, and prioritization, termed the “3P framework”. Prediction involves forecasting bloom risks and identifying environmental thresholds for early warning systems, and assessing the toxicity of cyanotoxins. Prevention emphasizes a proactive reduction in nutrient inputs, hydrological interventions, and biomanipulation for cyanobacterial blooms, as well as the prevention of exposure to cyanotoxins via contaminated drinking water or food, e.g., removing cyanotoxins during drinking water treatment. AI can help to integrate existing data to better understand the mechanisms for the occurrence/maintenance of cyanobacterial blooms, early warning systems, prevention and control, as well as the toxicity of cyanotoxins. Prioritization allocates limited resources to high-risk waters and cost-effective interventions for the protection of biodiversity and human health. Together, the 3P framework provides a proactive, evidence-based strategy for cyanobacterial risk management. Also, translating science and technology into practice requires closer collaboration among scientists, engineers, policymakers, regulators, industry, and the public. Therefore, in future, more studies and coordinated multi-disciplinary, multi-sectoral, and multi-stakeholder efforts are needed for the prevention, mitigation, and control of cyanobacterial blooms and cyanotoxin risk in the context of global climate change.

## 6. Conclusions

All of these studies have contributed to extending the knowledge on the complex ecology and toxicology of cyanobacteria and cyanotoxins. Much research on cyanobacteria and cyanotoxins has benefited from international collaborations, connecting laboratories with specialized expertise, e.g., ecology, environmental science and engineering, analytical chemistry, biochemistry, molecular biology, and toxicology. This is also the case with this Special Issue, “Ecology and Toxicology of Cyanobacteria and Cyanotoxins”, published in the journal *Toxins*. There are still many issues that require further research. We hope that this collection will attract a broad scientific audience and inspire new ideas and research programs at the intersection of cyanobacterial ecology, health, and industrial applications, which will advance the achievement of the Sustainable Development Goals (SDGs) through nature-based solutions (NbSs) and policies (NbPs), and ecosystem technology, under the “One Health” framework. Achieving this will require sustained multi-disciplinary, multi-sectoral, and multi-stakeholder collaboration, alongside stronger partnerships between scientists, engineers, policymakers, regulators, industry, and the public to translate research into actionable solutions.

## Figures and Tables

**Table 1:** Cyanotoxins, structures, biosynthetic genes, toxicity, and guideline values (GVs) [10,11,12,13,14,15,16,17].

Cyanotoxins	Toxin Types	Structure Types	Chemical Structure	Variants Number	Biosynthetic Genes	LD_50_ (i.p., Mice, μg/kg bm)	LD_50_ (Oral, Mice, μg/kg bm)	NOAEL (Oral, Mice, μg/kg bm)	LOAEL (Oral, Humans, μg/kg bm)	WHO GVs for Water (μg/L)	Main Toxic Effects	Toxic Mechanisms
Aplysiatoxins (APTXs)	Dermatotoxins	Phenolic bislactones	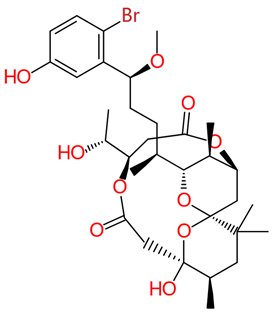	5		107–300					Dermatotoxic	Activation of protein kinase C (PKC)
Anatoxins (ATXs)	Neurotoxins	Alkaloids	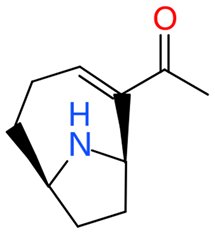	14	*anaA-H*	200–375	>5000	98		30 ^a^; 60 ^b^	Neurotoxic	Agonist of nicotinic acetylcholine receptors
β-N-methylamino-L-alanine (BMAA)	Neurotoxins	Diamino acids	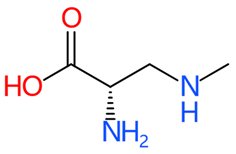	4							Neurotoxic	Stimulation of glutamate receptors
Cylindrospermopsins (CYNs)	Cytotoxins	Alkaloids	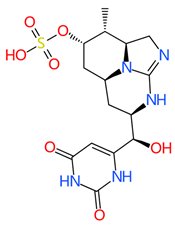	5	*cyrA-O*, *aoaA-C*	200–2100	4400–6900	30		0.7 ^c^; 3 ^d^; 6 ^e^	Hepatotoxic	Inhibition of protein synthesis
Guanitoxin (GNTX), formerly anatoxin(S) (ATX(S))	Neurotoxins	Organophosphate	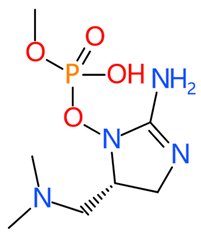			20–40					Neurotoxic	Inhibition of acetyl-cholinesterase
Lipopolysaccharides (LPSs)	Irritants	LPSs		3							Endotoxic	Activation of Toll-like receptor 4 (TLR4)
Lyngbyatoxins (LTXs)	Dermatotoxins	Alkaloids	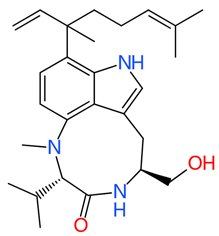	>8	*ltxA-D*	250					Dermatotoxic	Activation of PKC
Microcystins (MCs)	Hepatotoxins	Cyclic peptides	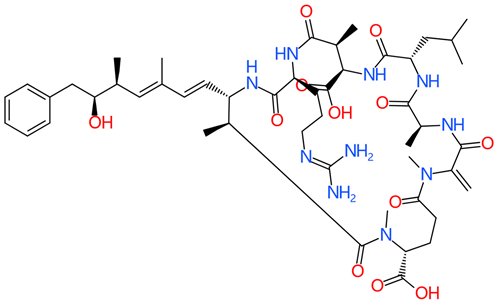	310	*mcyA-J*	25–1000	5000–10,900	40		1 ^c^; 12 ^d^; 24 ^e^	Hepatotoxic, carcinogenic	Inhibition of protein phosphatases (PP1, PP2A)
Nodularins (NODs)	Hepatotoxins	Cyclic peptides	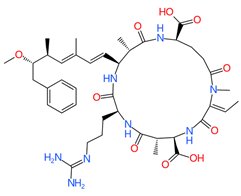	16	*ndaA-I*	30–70					Hepatotoxic, carcinogenic	Inhibition of PP1, PP2A
Saxitoxins (STXs)	Neurotoxins	Alkaloids	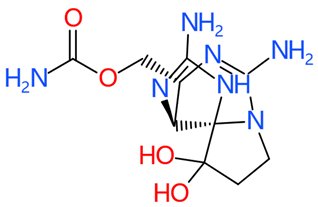	>60	*sxtA-Z*	5–65	263		1.5	3 ^f^; 30 ^g^	Neurotoxic	Blockage of voltage-gated sodium ion (Na+) channels

^a^ provisional short-term drinking-water health-based reference value (HBRV); ^b^ provisional recreation water HBRV; ^c^ provisional chronic (lifetime) drinking-water guideline value (GV); ^d^, provisional short-term drinking-water GV; ^e^ provisional recreation water GV; ^f^ acute drinking-water GV; ^g^ recreation water GV. bm, body mass; LOAEL, lowest observed adverse effect level; NOAEL, no observed adverse effect level; WHO, World Health Organization.

**Table 2:** Summary of the papers published in the Special Issue “Ecology and Toxicology of Cyanobacteria and Cyanotoxins”, published in the journal *Toxins*.

No.	Authors	Titles	Article Types	Weblinks
1	Lin et al. (2021)	Microcystin-Induced Immunotoxicity in Fishes: A Scoping Review	Review	https://doi.org/10.3390/toxins13110765
2	Hu et al. (2021)	Temporal Patterns of Bacterial and Viral Communities during Algae Blooms of a Reservoir in Macau	Article	https://doi.org/10.3390/toxins13120894
3	Dulić et al. (2022)	Assessment of Common Cyanotoxins in Cyanobacteria of Biological Loess Crusts	Article	https://doi.org/10.3390/toxins14030215
4	Swartzendruber et al. (2022)	Lipopolysaccharide from the Cyanobacterium *Geitlerinema* sp. Induces Neutrophil Infiltration and Lung Inflammation	Article	https://doi.org/10.3390/toxins14040267
5	Pesnya et al. (2022)	Genotoxicity of Natural Water during the Mass Development of Cyanobacteria Evaluated by the Allium Test Method: A Model Experiment with Microcosms	Article	https://doi.org/10.3390/toxins14050359
6	Jiang et al. (2022)	Gill Junction Injury and Microbial Disorders Induced by Microcystin-Leucine Arginine in *Lithobates catesbeianus* Tadpoles	Article	https://doi.org/10.3390/toxins14070479
7	Zhang et al. (2022)	Transcriptome Analysis Reveals the Algicidal Mechanism of *Brevibacillus laterosporus* against *Microcystis aeruginosa* through Multiple Metabolic Pathways	Article	https://doi.org/10.3390/toxins14070492
8	Saha et al. (2022)	Subchronic Oral Cylindrospermopsin Exposure Alters the Host Gut Microbiome and Is Associated with Progressive Hepatic Inflammation, Stellate Cell Activation, and Mild Fibrosis in a Preclinical Study	Article	https://doi.org/10.3390/toxins14120835
9	Yang et al. (2023)	Integration of Transcriptomics and Microbiomics Reveals the Responses of *Bellamya aeruginosa* to Toxic Cyanobacteria	Article	https://doi.org/10.3390/toxins15020119
10	Nugumanova et al. (2023)	Freshwater Cyanobacterial Toxins, Cyanopeptides and Neurodegenerative Diseases	Review	https://doi.org/10.3390/toxins15030233
11	Liu et al. (2023)	Gut Microbiota of Freshwater Gastropod (*Bellamya aeruginosa*) Assist the Adaptation of Host to Toxic Cyanobacterial Stress	Article	https://doi.org/10.3390/toxins15040252
12	Chatterjee and More (2023)	Cyanobacterial Harmful Algal Bloom Toxin Microcystin and Increased *Vibrio* Occurrence as Climate-Change-Induced Biological Co-Stressors: Exposure and Disease Outcomes via Their Interaction with Gut-Liver-Brain Axis	Review	https://doi.org/10.3390/toxins15040289
13	Zhan and Gong (2024)	Mutations at Two Key Sites in PP2A Safeguard *Caenorhabditis elegans* Neurons from Microcystin-LR Toxicity	Article	https://doi.org/10.3390/toxins16030145

## Abbreviations

The following abbreviations are used in this manuscript:

AETX	aetokthonotoxin
AI	artificial intelligence
ALF	acute liver failure
ANOSIM	analysis of similarity
ATX	anatoxins
BLCs	biological loess crusts
bm	body mass
BMAA	β-N-methylamino-L-alanine
C	carbon
CKD	chronic kidney disease
CVD	cardio-vascular disease
CyanoHABs	cyanobacterial harmful algal blooms
CYN	cylindrospermopsin
DAMP	damage-associated molecular pattern
DEG	differentially expressed gene
DRG	down-regulated gene
Fe	ferrum
GNTX	guanitoxin
GSH	glutathione
H&E	hematoxylin and eosin
HE	hepatic encephalopathy
IFN	interferon
Ig	immunoglobulin
IL	interleukin
LC-MS/MS	liquid chromatography-tandem mass spectrometry
LOAEL	lowest observed adverse effect level
LPS	lipopolysaccharide
MASLD	metabolic dysfunction-associated steatotic liver disease
MC	microcystin
N	nitrogen
NbP	nature-based policy
NbS	nature-based solution
NOAEL	no observed adverse effect level
NOD	nodularin
NTB	non-traditional biomanipulation
P	phosphorus
PAMP	pathogen-associated molecular pattern
PCoA	principal coordinate analysis
PMOS	poly-endocrine metabolic ovarian syndrome
PP	protein phosphatase
ROS	reactive oxygen species
S	sulfur
SDG	Sustainable Development Goal
SOD	superoxide dismutase
STX	saxitoxin
T2DM	type 2 diabetes mellitus
T&O	taste and odor
TB	traditional biomanipulation
TLR	Toll-like receptor
TNF	tumor necrosis factor
RDA	redundancy analysis
URG	up-regulated gene
UV	ultraviolet
WHO	World Health Organization
